# Understanding the microbial basis of body odor in pre-pubescent children and teenagers

**DOI:** 10.1186/s40168-018-0588-z

**Published:** 2018-11-29

**Authors:** Tze Hau Lam, Davide Verzotto, Purbita Brahma, Amanda Hui Qi Ng, Ping Hu, Dan Schnell, Jay Tiesman, Rong Kong, Thi My Uyen Ton, Jianjun Li, May Ong, Yang Lu, David Swaile, Ping Liu, Jiquan Liu, Niranjan Nagarajan

**Affiliations:** 1 Procter & Gamble Singapore Innovation Center, Singapore, 138547 Singapore; 2 Computational and Systems Biology, Genome Institute of Singapore, Singapore, 138672 Singapore; 3 Procter & Gamble Mason Business Center, Mason, OH 45040 USA; 4 Procter & Gamble Sharon Woods Innovation Center, Sharonville, OH 45241 USA; 5 Yong Loo Lin School of Medicine, National University of Singapore, Singapore, 119228 Singapore

## Abstract

**Background:**

Even though human sweat is odorless, bacterial growth and decomposition of specific odor precursors in it is believed to give rise to body odor in humans. While mechanisms of odor generation have been widely studied in adults, little is known for teenagers and pre-pubescent children who have distinct sweat composition from immature apocrine and sebaceous glands, but are arguably more susceptible to the social and psychological impact of malodor.

**Results:**

We integrated information from whole microbiome analysis of multiple skin sites (underarm, neck, and head) and multiple time points (1 h and 8 h after bath), analyzing 180 samples in total to perform the largest metagenome-wide association study to date on malodor. Significant positive correlations were observed between odor intensity and the relative abundance of *Staphylococcus hominis*, *Staphylococcus epidermidis*, and *Cutibacterium avidum*, as well as negative correlation with *Acinetobacter schindleri* and *Cutibacterium* species. Metabolic pathway analysis highlighted the association of isovaleric and acetic acid production (sour odor) from enriched *S. epidermidis* (teen underarm) and *S. hominis* (child neck) enzymes and sulfur production from *Staphylococcus* species (teen underarm) with odor intensity, in good agreement with observed odor characteristics in pre-pubescent children and teenagers. Experiments with cultures on human and artificial sweat confirmed the ability of *S. hominis* and *S. epidermidis* to independently produce malodor with distinct odor characteristics.

**Conclusions:**

These results showcase the power of skin metagenomics to study host-microbial co-metabolic interactions, identifying distinct pathways for odor generation from sweat in pre-pubescent children and teenagers and highlighting key enzymatic targets for intervention.

**Electronic supplementary material:**

The online version of this article (10.1186/s40168-018-0588-z) contains supplementary material, which is available to authorized users.

## Introduction

Body odor in the animal kingdom frequently plays an important role in defense against predators and survival [1–3]. In humans, olfactory cues are believed to have a role in kinship detection and mate selection [4]. Despite these functions, body odor generally has a strong social stigma attached to it, with its social and psychological impact on affected subjects not being fully understood.

Body odor in humans is believed to be primarily mediated by bacterial decomposition of naturally secreted, non-odorous constituents of sweat, especially fatty acids, branched-chain aliphatic amino acids, glycerol, and lactic acid originating from eccrine, apocrine, and sebaceous glands [5–8]. In studies in adults using marker gene sequencing (16S rRNA) and other approaches, a range of bacteria but primarily *Corynebacterium* species have been associated with malodor [6, 9, 10]. Additionally, specific bacterial enzymes and pathways that act on non-odorous proteinaceous precursors or produce odor molecules such as acetic acid, volatile fatty acids, sulfanyls, and thioalcohols have also been identified using culture-based approaches [5–7, 10, 11]. Significant differences exist, however, between odor-related physiology and the skin microbiota of adults and non-adults. For example, apocrine glands (mostly found in bodily locations with hair follicles, e.g., axilla) are inactive until stimulated by hormonal changes in puberty, and unlike eccrine glands which secrete continuously, apocrine glands secrete periodically [12]. Eccrine sweat (clear, odorless, pH of 4.0–6.8, composed of 98–99% water but also containing sodium chloride, fatty acids, lactic acid, citric acid, ascorbic acid, urea, and uric acid) and apocrine sweat (odorless, pH 6.0–7.5, containing water, proteins, carbohydrates, lipids, and steroids) have distinct compositions and distribution (all body sites vs inside hair follicles) [13]. Furthermore, sebaceous glands that secrete an odorless oily matter called sebum into hair follicles (pH 4.5–5.5) have increased activity during puberty [14]. Thus, body odor is generally considered significant only in post-pubescent subjects and adults and has primarily been studied within axilla where apocrine glands outnumber eccrine glands by a factor of 10.

Most studies on malodor have been conducted in temperate climates and on subjects from Europe and North America. Asians, particularly those of East Asian heritage, are believed to be genetically predisposed to lower malodor [15, 16]. However, tropical environments that are hot and humid can increase sweat production and associated incidence of malodor. Based on extensive surveys in a Southeast Asian context, we identified that body odor is indeed a significant issue in pre-pubescent children and teenagers, both in terms of parent-reported scores as well as expert evaluations (Additional file 1: Table S1). The impact of malodor during childhood on the emotional, social, and psychological well-being of subjects is an uncharted field but it is likely to be of greater concern than adult malodor. Furthermore, as apocrine gland secretion is inactive in children, the host-microbial metabolic interactions that lead to malodor remains unexplained.

To address this subject, we recruited a cohort of pre-pubescent children and teenagers in the Philippines who were assessed by a professional perfumer for recording odor intensity (range 0–100) and character (e.g. sweet, greasy, rancid, sour, hircine, sulfur), in addition to an assessment by their parents (Additional file 2: Table S2). The study design incorporated sampling from multiple body sites (neck, underarm, head) associated with body odor, as well as multiple time points after bath and outdoor activity, to systematically understand the microbial basis of body odor in these individuals. In total, 180 samples were subjected to deep whole-metagenome sequencing analysis, allowing us to study bacterial, viral, and eukaryotic community members and their functional association with malodor. Multi-modal analysis, combining regression to odor intensity and differential signatures before and after outdoor activity, was used to identify microbial taxa and functions associated with malodor in children (5–9 years) and teenagers (15–18 years). These signatures have some overlap (e.g., *S. hominis*) but also distinct aspects from what has been reported in adult cohorts. Overall, our study highlights the importance of moving from a “bag of genes” to a “bag of genomes” analysis of metagenomes to comprehensively study host-microbial metabolic interactions.

## Results

### Distinct odor intensities and characteristics are associated with different body parts in children and teenagers

**Fig. 1:**
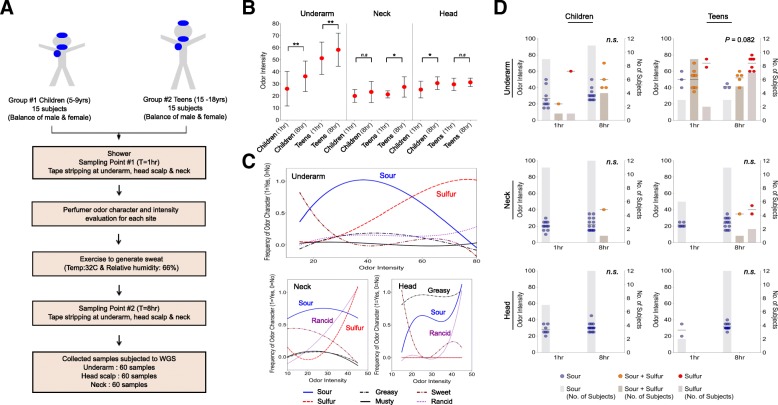
Study design and relationships between odor intensity, odor characteristics, age, and body sites. **a** Schema detailing the study target groups, sample numbers, and collection timepoints. **b** Odor intensity distributions across body sites, before (1 h) and after (8 h) exercise and across age groups (children and teens; **p-value < 0.05, *p-value < 0.1). **c** Trendlines depicting the relationship between different odor characteristics and odor intensity across body sites (1 = present, 0 = absent). **d** Relative distribution of sour and sulfur odor in subjects before (1 h) and after (8 h) exercise across age groups and body sites. Dots mark odor intensity as shown on the left axis while bar-charts indicate the number of subjects as shown on the right axis

Our study was based on two distinct age groups of individuals, pre-pubescent children (5–9 years) and teenagers (15–18 years), allowing for a more detailed investigation of the microbial basis of malodor in early stages of life (see the “Materials and methods” section, Fig. 1a, Additional file 2: Table S2). While the underarm (axilla) has been the primary site of investigations for malodor, consumer studies suggested that malodor from the neck (nape) and head (mid-scalp) areas were also regions of concern for families and were therefore included in this study (Additional file 1: Table S1). Subjects were assessed at two timepoints, separated by 7 h of rest and exercise to generate sweat, providing a well-suited experimental setup to account for inter-host variability and investigate how microbes convert sweat precursors into malodor (Fig. 1a). In addition, the first timepoint was taken 1 h after shower, providing a standardized baseline for all individuals. Samples were taken on the same day and site in the Philippines (Manila), with a relatively constant ambient temperature at 32 °C and humidity at 66%.

In addition to parent-assessed odor groups (Additional file 2: Table S2), a single professional perfumer assessed odor intensity and character in all subjects, sites, and at all time points. As expected, odor intensity as measured by the professional perfumer was significantly higher in the underarms of children and teenagers after exercise (Wilcoxon test *p*-value < 0.05) (Fig. 1b). Marginally significant increases in odor intensities were also detected in the neck and head regions of teenagers and children, respectively (Wilcoxon test *p*-value < 0.1). Teenagers had higher odor intensity than children in the underarm before (Wilcoxon test *p*-value = 2.4 × 10^− 4^) and after (Wilcoxon test *p*-value = 2.6 × 10^− 4^) exercise, and in general, odor intensities were lower in the neck and head regions compared to the underarm region. In terms of odor characteristics, the underarm and neck regions were marked by sour and sulfur odors in the medium and high odor intensity ranges, respectively (Fig. 1c). In contrast, the head region was primarily characterized by a greasy odor, highlighting that different regions of the body are likely to have different microbial metabolism contributing to malodor. Analyzing odor characteristics by age group and timepoint revealed that while children are primarily defined by sour or sour+sulfur odor characteristics, teenagers are more likely to have a sour+sulfur characteristic that shifts to a sulfur-dominant characteristic after exercise (Fig. 1d). This property however seems to be specific to the underarm, and in the neck and head regions, sour odor is dominant at both time points in children and teenagers (Fig. 1d). These observations provide the essential backdrop for our analysis of microbial contributions to malodor in the following sections.

### Features of skin microbiome across age groups and in association with malodor

To study the taxonomic and functional composition of the skin microbiome in our cohort, samples were collected using a newly established tape stripping protocol [17], providing sufficient DNA for constructing shotgun metagenomic libraries for 100% of our samples and none of the control samples (collection and laboratory controls). Deep sequencing on the Illumina platform was used to characterize the skin microbiome samples for all individuals, sites, and timepoints (180 samples, ~ 69 million reads on average; Additional file 2: Table S2). On average, the reads were of high quality (> Q30) with more than 98% of reads being usable after quality filtering (Additional file 2: Table S2). Leveraging the metagenomic data for cross-kingdom analysis, we noted that different body sites exhibited different kingdom level distributions (Additional file 1: Figure S1). Bacteria were more predominantly present in underarm samples (Wilcoxon test *p*-value < 3 × 10^− 4^), while eukaryotes were comparatively more abundant on children’s heads (Wilcoxon test *p*-value < 2 × 10^− 9^), and neck samples had a uniquely higher relative proportion of viruses (Wilcoxon test *p*-value < 7 × 10^− 3^). The increased abundance of viruses and eukaryotes in the head and neck regions is similar to what has been observed in nearby sites (retroauricular crease, occiput) in adults, indicative of the unique niches that they offer [18].

**Fig. 2:**
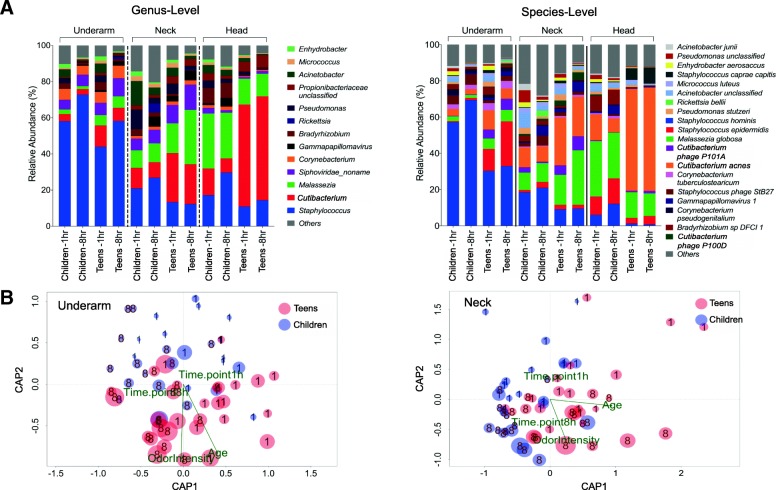
Association between skin microbiome, odor intensity, age, and time of sampling. **a** Relative abundance of skin microbes (> 0.1%) across sites at genus and species level. Data are represented as the mean relative abundance within a group. **b** Ordination biplots for canonical analysis of principal coordinates (CAP) illustrating the strength of associations between odor intensity, age, sampling time, and the skin microbiome. CAP analysis is based on the Bray-Curtis dissimilarity. Size of each dot represents the relative odor intensity score in each sample and the number overlapping each dot indicates samples collected before (1) or after (8) exercise

Specific microbiome signatures emerged for each body site at the bacterial species and genus level as well (Additional file 1: Figures S2, S3), despite the presence of clear differences between the microbiomes of teenagers and children (Fig. 2a, Additional file 1: Figure S4). For example, *Staphylococcus hominis* is the most abundant bacterial species in the underarm, despite notable differences between children and teenagers (70% vs 37%; Wilcoxon test *p*-value < 5 × 10^− 4^). Similarly, while the increased presence of *M. globosa* and *C. acnes* distinguishes the neck and head from the underarm, children have relatively more *M. globosa* on their head while teenagers are dominated by *C. acnes* (Fig. 2a, Additional file 1: Figure S3, S4). These differences correlate with changes in apocrine gland activity during puberty and can serve as the basis for differences in malodor characteristics between children and teenagers. Additionally, all subjects had a much lower underarm abundance of *Corynebacterium* species than what has been observed in adults [9] suggesting that distinct species may play a role in malodor in these age groups.

Canonical analysis of principal coordinates of variation of the skin microbiome in different body sites in relation to age group, gender, and timepoints (1 h or 8 h) further illustrates the strength of associations between odor intensity, age, sampling time, and the skin microbiome (Fig. 2b). In the underarm, the specific age groups and time of sampling correlate well with odor intensity (*p*-value < 0.001) and less so with gender (*p*-value < 0.05) as a function of skin microbiome composition. This pattern is also observed in the neck but is not significant on the head, where there is a stronger association with gender (Additional file 1: Figure S5). Microbiome composition in the head area is also unique in that it does not seem to shift noticeably after exercise. One possibility for this is the limited impact of a shower on the scalp microbiome compared to other sites, with microbes residing in the hair follicle making the scalp microbiome more stable and resilient.

### *Staphylococcus* species strongly correlate with odor intensity in children and teenagers

We noted that children belonging to parent-defined categories (“malodor” and “no malodor”) did not exhibit significantly different odor intensity as defined by a professional perfumer, across age groups and timepoints (Additional file 1: Figure S6), potentially highlighting the challenges of relying solely on consumer perception for such analysis. The “malodor” groups however did show a tendency to exhibit higher odor intensity and this difference may reach statistical significance with larger sample sizes. Instead of doing categorical analysis in this study, we looked for microbial taxa and functions that associated directly with perfumer-defined odor intensity (see the “Materials and methods” section). In addition, the availability of data from two timepoints, before and after exercise, was further used to identify microbial taxa and functions that change longitudinally in association with increased sweat and odor production (see the “Materials and methods” section, Additional file 1: Figure S7, Table 1).

**Table 1:** List of microbes at the species level that show significant correlation with odor intensity in at least one age group

Species	Youth				Children			Teenagers				
	Spearman’s *ρ*		Wilcoxon		Spearman’s *ρ*		Wilcoxon	Spearman’s *ρ*			Wilcoxon	
	*ρ*	*p*-value	*p*-value		*ρ*	*p*-value	*p*-value	*ρ*	*p*-value		*p*-value	
Underarm												
*Staphylococcus epidermidis*	0.67	5 × 10^−7^	↑	0.04	0.44	n.s.	↑	n.s.	0.40	n.s.	↑	n.s.
*Cutibacterium avidum*	0.63	5 × 10^−6^	↑	n.s.	0.52	n.s.	↑	n.s.	0.31	n.s.	↑	n.s.
*Corynebacterium pseudogenitalium*	0.44	10^−2^	↓	n.s.	0.42	n.s.	↓	n.s.	0.13	n.s.	↑	n.s.
*Corynebacterium tuberculostearicum*	0.38	0.06	↓	n.s.	0.35	n.s.	↓	n.s.	0.18	n.s.	↑	n.s.
*Malassezia globosa*	0.53	7 × 10^−4^	↓	n.s.	0.37	n.s.	↓	n.s.	0.33	n.s.	↑	n.s.
Neck												
*Staphylococcus hominis*	0.32	n.s.	↑	n.s.	0.65	10^−2^	↑	n.s.	0.09	n.s.	↑	n.s.
*Staphylococcus phage StB27*	0.49	0.02	↑	n.s.	0.77	10^−4^	↑	n.s.	0.19	n.s.	↑	n.s.
*Cutibacterium granulosum*	− 0.45	0.03	↓	0.06	− 0.48	n.s.	↓	n.s.	− 0.38	n.s.	↓	n.s.
*Acinetobacter schindleri*	− 0.43	0.03	↓	0.07	− 0.47	n.s.	↓	n.s.	− 0.30	n.s.	↓	n.s.
*Cutibacterium acnes*	− 0.28	n.s.	↓	n.s.	− 0.54	0.08	↓	n.s.	− 0.21	n.s.	↓	n.s.

All reported *p*-values are adjusted for multiple hypotheses testing through FDR analysis (*p*-value < 0.1) and with Spearman’s *ρ* > 0.2. Arrows indicate the directionality of change in relative abundance after exercise (up: ↑ or down: ↓). The “youth” group combines children and teenagers

n.s.: not significant (*p*-value > 0.1)

In general, consistent trends were observed between the cross-sectional analysis and longitudinal analysis, where taxa that were positively correlated with malodor also increased in abundance after exercise (Table 1). The age group-specific analysis identified key differences between the groups; though in some cases, subtle but consistent trends were amplified by combining data across age groups (denoted as “Youth”). For example, in the underarm, *Staphylococcus epidermidis* showed the strongest association with malodor when both age groups were jointly analyzed (Additional file 1: Figure S7, Table 1), though the trend was consistently seen in each age group and in timepoints before and after exercise. In contrast on the neck, *Staphylococcus hominis* was associated with malodor intensity, but only in children (*ρ* = 0.65) and with low correlation observed in teenagers (*ρ* = 0.09; Additional file 1: Figure S7, Table 1). Interestingly, a *Staphylococcus* phage was also observed to be positively correlated with malodor, potentially driven by the association of its host species and highlighting the importance of phage-bacteria interactions on the skin [19, 20]. This analysis further emphasizes the important role that *Staphylococcus* species play in malodor in children and delineates their specific contributions in different body sites and age groups [5, 8].

A *Cutibacterium* species (*C. avidum*) was the only other organism with a consistent association with higher malodor, specifically in the underarm, though its significance remains unclear. Other *Cutibacterium* species (*C. granulosum*, *C. acnes*) were negatively associated with malodor in the neck region (Table 1), potentially due to inter-bacterial competition with *Staphylococcus* species [21, 22]. *Corynebacterium* species (*C. pseudogenitalium* and *C. tuberculostearicum*) were positively correlated with odor intensity in the underarm, but with a consistent increase in relative abundance after exercise only being seen in teenagers (Table 1). As *Corynebacterium* species are known to produce sulfur odorants [23, 24], this observation could be a reflection of the fact that the underarm skin microbiome pre-puberty lacks the precursors from apocrine secretion to support their metabolism into a sulfurous malodor.

Interestingly, *Acinetobacter* species (specifically *A. schindleri*) were enriched in the neck area of children with lower malodor intensity and also at the 1 h time point. The pathways involved in these species in mitigating malodor perception are unclear but one possible mechanism is through competition with other microbes via degradation of malodor precursors into aliphatic molecules [25]. In agreement with our earlier analysis (Additional file 1: Figure S5), no microbes were found to be malodor-associated in the head area.

Overall, these analyses highlight the varied microbial associations with body odor and that different distributions of key microbial factors and odor components across individuals, body sites, age groups, and timepoints may play a role in odor production. Notably, genus level analysis of the microbiome was unable to identify key associations in the underarm (Additional file 1: Table S3), highlighting the importance of species differences and the strengths of shotgun metagenomic analysis for studying malodor-associated microbes. In particular, our results highlight the dominant role that *S. epidermidis* may be playing in underarm malodor with sour characteristics, that we frequently noted in children and to an extent in teenagers (Fig. 1d). The transition through puberty leads to an increasingly sulfur dominated odor characteristic and shifts in *Staphylococcus* and *Corynebacterium* activity may contribute to this [5]*.* We further explore these relationships in the next section through analysis of malodor associated pathways in the skin microbiome.

### Identification of isovaleric and acetic acid production as a key malodor-associated pathway in children and teenagers

As part of the study design, body sweat was collected from the subjects, pooled, and used for various analyses (high-performance liquid chromatography (HPLC), liquid chromatography and gas chromatography-mass spectrometry (LC/GCMS)) to identify odor precursors that may be differentially present in children and teenagers (see the “Materials and methods” section). This analysis showed that lactic acid was the most abundant precursor in children and teenagers followed by glycerol (more abundant in teenagers), isoleucine, and leucine (Additional file 1: Table S4). Pooled sweat was then incubated for 24 h to measure the production of malodor-associated molecules using GC-olfactometry (GC-O) and GCMS analysis (Additional file 1: Figure S8 and Additional file 2: Table S5). Overall, children and teenagers revealed similar GCMS and GC-O profiles with teenagers being more abundant for the dominant compounds—acetic acid and isovaleric acid. These two potent odorants have acidic/sour smell character suggesting that the observed “sourness bouquet” in our subjects is largely due to these two odorants.

Based on this information, we then went on to do a functional analysis to discover malodor-associated genes and pathways in vivo and in a data-driven manner. We used a similar approach as used for the taxonomic analysis to identify genes and an initial set of pathways correlated with malodor intensity and those that are overrepresented after exercise and sweat production (see the “Materials and methods” section; Additional file 2: Table S6). Significantly associated genes were then aggregated in pathways to understand their contribution to the production of malodor-causing compounds. We first used a “bag of genes” approach, as is commonly used in metagenomics, aggregating gene abundances without accounting for the taxonomic origin of reads to find malodor associations. Mapping the identified genes onto pathways, however, did not reveal consistent associations within a pathway (e.g., Additional file 1: Figure S9), likely due to the presence of multiple related species (e.g., *S. epidermidis* and *S. hominis*) with distinct contributions to malodor. In contrast, a “bag of genomes” analysis that incorporated the species identity of genes highlighted several significant and consistent pathway associations and corresponding species contributions (Additional file 2: Table S7).

**Fig. 3:**
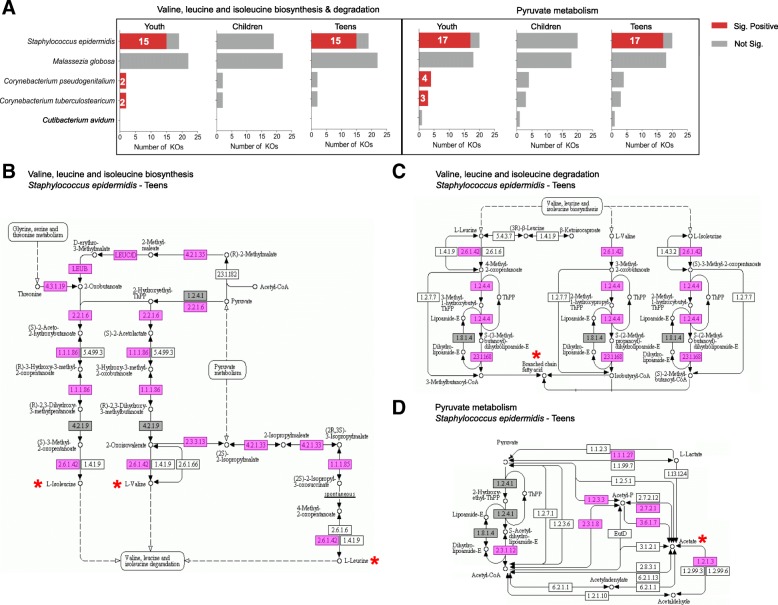
Pathways and enzymes associated with sour odor-producing compounds in the underarm. **a** Number of enzymes (KOs) encoded by malodor-associated microbes in the underarm region for “valine, leucine, and isoleucine biosynthesis/degradation” and “pyruvate metabolism” KEGG pathways. Numbers in the bars indicate the number of KOs where the relative abundance is significantly correlated with odor intensity. **b**–**d** Corresponding KEGG pathway diagrams, with pink boxes indicating significant positively correlated KOs in *S. epidermidis* among teenagers and gray boxes indicating enzymes that are present but do not have a significant correlation with odor intensity. Odor producing compounds and their precursors are marked with a star (*)

**Fig. 4:**
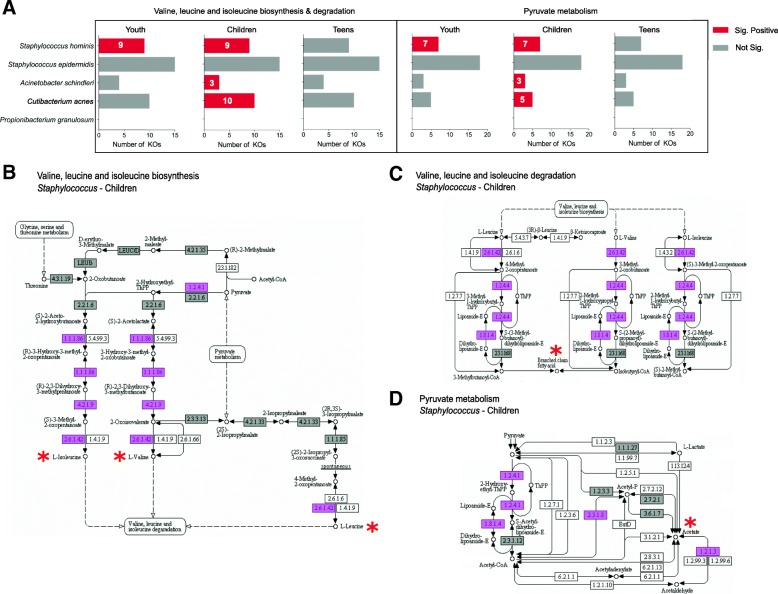
Pathways and enzymes associated with sour odor-producing compounds in the neck. **a** Number of enzymes (KOs) encoded by malodor-associated microbes in the neck region for “valine, leucine, and isoleucine biosynthesis/degradation” and “pyruvate metabolism” KEGG pathways. Numbers in the bars indicate the number of KOs where the relative abundance is significantly correlated with odor intensity. **b**–**d** Corresponding KEGG pathway diagrams, with pink boxes indicating significant positively correlated KOs in *Staphylococcus* species among children and gray boxes indicating enzymes that are present but do not have a significant correlation with odor intensity. Odor producing compounds and their precursors are marked with a star (*)

Particularly in the underarm, pyruvate metabolism and branched-chain amino acid metabolism pathways were enriched for genes in *S. epidermidis* that were significantly positively correlated with malodor, consistent with our taxonomic analysis (Fig. 3a)*.* Enzymes derived from *S. epidermidis* are involved in a cascade of multiple metabolic pathways leading to the production of both sour odor-associated acetic acid and isovaleric acid. Lactic acid and glycerol are converted to pyruvate in the glycolysis pathway. In the presence of pyruvate, enzymes responsible for the biosynthesis and subsequently the degradation of leucine, valine, and isoleucine to yield the branched-chained fatty acid isovaleric acid (e.g., the key enzymes acetolactate synthase (EC:2.2.1.6) and branched-chain amino acid transaminase (EC:2.6.1.42)) were found to be significantly associated with malodor (Fig. 3b, c). In an independent pathway, enzymes involved in oxidative degradation of pyruvate aliphatic carboxylates to acetyl-CoA (Swanson Conversion) in a process that is key to the production of acetic acid and sour odor were found to be malodor-associated in *S. epidermidis* (Fig. 3d; e.g., pyruvate dehydrogenase (EC:1.2.4.1)). These results agree with previously reported in vitro studies [10]. Similar pathways were also associated with malodor in the neck, but this time, the associations were seen primarily with *S. hominis* genes and more significantly in children (Fig. 4).

3-Methyl-3-sulfanylhexanol (3M3SH), 3-methyl-2-hexenoic acid (3M2H), and 3-methyl-3-hydroxy-hexanoic acid (HMHA) are known odorants responsible for axilla odor in adults [7]. For the production of 3-methyl-3-sulfanylhexanol (3M3SH), which is associated with malodor with sulfur characteristics, we observed a positive association with both *Staphylococcus* species (*S. hominis* and *S. epidermidis*; Additional file 1: Figure S10). Previous in vitro observations have demonstrated the ability of *S. hominis*, but not *S. epidermidis*, to generate 3MS3H [5]. Our metagenomic results indicate the presence of cystathionine beta-lyase in *S. epidermidis*, suggesting that other than *S. hominis*, *S. epidermidis* strains isolated in Asian populations could also generate 3M3SH. Further in vitro experiments are needed to validate this possibility. For 3M2H and HMHA, our GCMS analysis detected no or minimal presence of these odorants. In addition, our metagenomic data did not reveal the presence of *Corynebacterium striatum* and the aminoacylase enzyme (agaA; in both children and teenagers) which have been shown to mediate the production of HMHA [23]. This data indicates that these molecules are unlikely to be the main drivers of malodor in our cohorts, compared to their key role in adults [14].

**Fig. 5:**
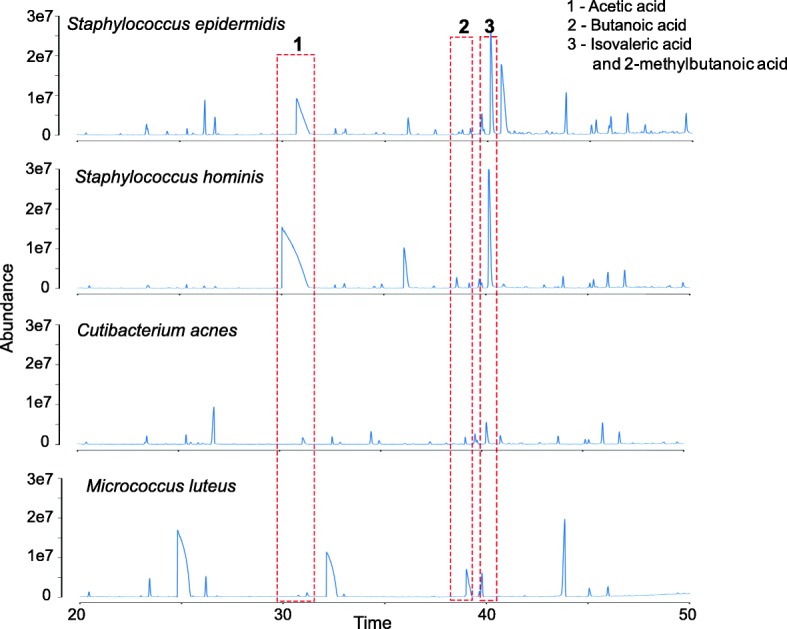
Capability to transform precursors in sweat into malodor-associated compounds in different skin microbes. GCMS results from single bacterium inoculation for 24 h in sweat collected from subjects. Note that only *S. epidermidis* and *S. hominis* produce significant amounts of acetic acid and isovaleric acid, in agreement with the associations observed in vivo. The culture experiments were conducted under aerobic conditions

To test the association of *Staphylococcus* species with sour odor characteristics, we cultured isolates in sterilized sweat pools obtained from subjects enrolled in the study and measured the production of various malodor compounds using GCMS analysis (see the “Materials and methods” section). While both the *Staphylococcus* species were able to produce significant quantities of acetic acid and isovaleric acid, other species found commonly on the skin such as *C. acnes* and *M. luteus* did not (Fig. 5). These results highlight the primary role of *Staphylococcus* species in the production of acetic acid, isovaleric acid, and 3M3SH from odor precursors in sweat and in explaining the sour and sulfur odor characteristics observed in Asian children and teenagers.

## Discussion

Malodor is a phenotype that is well known to arise from specific interactions between host-derived odor precursors and the microbial metabolism that they support [6]. In this respect, this study serves as an ideal test-bed for exploring analytical approaches to uncover host-microbiome metabolic interactions that define a phenotype of interest. While malodor in adults and Caucasian populations has been extensively studied [5, 6, 9], malodor in Asians and particularly in children and teenagers has not received commensurate attention. Our investigations show that it is indeed a problem of consumer interest that impacts the quality of life of affected children, suggesting that its influence on the psychological well-being of children deserves further research.

In designing a study to identify malodor associated microbial species and pathways, several aspects need to be considered, including the possibility that there might be taxa that are slow growing but strongly associated with malodor, while others may only generate malodor when they are sufficiently abundant. Our design thus incorporated both longitudinal (using exercise to generate sweat and odor precursors) and cross-sectional aspects in multiple age groups to broadly capture different signals of association. Our data indicates that there are consistent signals from the longitudinal and cross-sectional analyses in most cases, except for *Corynebacterium* species and *M. globosa* in children’ underarms, whose apparent reduction in relative abundance with exercise (despite a positive correlation with malodor) may be due to their slower growth in comparison to *S. epidermidis*. Additionally, the data in this study highlights different microbial contributors to body odor in different age groups and body sites, e.g., *S. hominis* was found to have a strong association with malodor in the neck region for children, while the underarm region showed significant association with *S. epidermidis*. This is despite the abundance of *S. hominis* in the underarm region, suggesting that differences in sweat precursors from the neck and underarm regions play a role here and that *S. epidermidis* can have an outsized effect for malodor generation when the right precursors are present.

Prior work to study microbial contributions to axilla malodor has primarily emphasized the role of *Corynebacterium* species [7] in adults. Using 16S rRNA sequencing, these studies have shown that *Corynebacterium*, *Staphylococcus*, and *Cutibacterium* species are the dominant bacteria in the axilla region [9, 26–28]. Of these, *Corynebacterium* species were shown to be more abundant than *Staphylococcus* species in groups of individuals not using antiperspirants or deodorants [26]. Malodor in adult axilla was also typically found to be dominated by sulfur or rancid odor characteristics from 3M3SH, 3M2H, and HMHA generated mainly by *Corynebacterium* species [7, 9, 29]. In comparison, our study shows that *Corynebacterium* species are less abundant in children and teenagers and *Staphylococcus* species are more abundant. This difference may be in part due to the axilla microenvironment in children and teenagers being shaped by eccrine secretion. While technical differences in metagenome profiling could also affect these comparisons, we note that shotgun metagenomics and 16S rRNA profiling have shown good concordance in a previous study [17]. In addition, sour odor was found to be more common in children in this study, and correspondingly, our analysis has highlighted the central role of *Staphylococcus* species in body odor in children. Among *Staphylococcus* species, while most earlier studies have investigated the role of *S. haemolyticus* and *S. hominis* [5, 23], our work is the first to identify the role of *S. epidermidis* in underarm malodor in children. This has significant consequences in terms of interventions for malodor—while the main approach for adults uses antiperspirants to control malodor production by slow-growing *Cornybacterium* species, antimicrobials may be more appropriate for the fast-growing *Staphylococcus* species in children as antiperspirants have been observed to relatively enrich for them [26]. Additionally, as *S. epidermidis* is an important skin commensal [30], next-generation treatments that block the activity of specific enzymes identified in this study may be needed.

In terms of the influence of host genetics on malodor, several studies have described the association of ABCC11 alleles with body odor in adults and have shown that individuals carrying the GG/GA alleles have increased odor due to the generation of 3M3SH, 3M2H, and HMHA from apocrine sweat [15, 16, 31]. A majority of the Philippines population (~ 72 to 78%) carries the GG/GA allele, and hence, adults in the Philippines are more likely to be affected by malodor with sulfur/rancid characteristics [15, 32]. In pre-pubescent children, apocrine glands are not fully activated [12], and thus, eccrine-derived precursors could play a more significant role in the observed odor characteristics. Apocrine glands are comparatively more active in teenagers whose malodor profile is therefore expected to be more similar to adults. These expectations match with our observations of higher odor intensity in teenagers versus children and a switch to more sulfur characteristics in teenagers.

Our bioinformatic analysis in this study has further highlighted the challenges of a “bag of genes” approach to studying microbiome function with metagenomic data. Microbes exchange metabolites in a controlled fashion and assuming that multiple steps of a metabolic process can be done in different species in a “metagenome” may not always be appropriate [33, 34]. In addition, enzymes from different species that belong to the same enzyme class may have very different efficiencies and substrate specificities [35], e.g., explaining why *S. epidermidis* was associated with underarm malodor in children but *S. hominis* was not. This problem is exemplified by a recent report showing that metagenomic pipelines can highlight very different functional pathways using the same dataset [36]. Moving to a “bag of genomes” analysis could enable better interpretability of metagenomic datasets, but a significant limitation is the ability to identify different genes that all belong to the same genome. Enhanced clustering and assembly approaches enabled by advances in sequencing technology could help address these limitations in the future [37, 38].

To our knowledge, this is the first report to describe taxa that are negatively correlated with malodor but their mechanism of action remains unclear. These associations could be due to various reasons including (a) the production of compounds that mask malodor or have lower odor intensity (as proposed here for *P. avidum*), (b) microbial competition or inhibition leading to reduced growth of malodor-associated species (as has been described between *Malassezia* and *Staphylococcus* species [39]), and (c) metabolic processes that lead to key odor precursors being unavailable for generating malodor compounds. Culture experiments with human sweat as performed here provided a unique framework to realistically model precursor availability and highlight the contribution of *Staphylococcus* species for odor generation. Further experiments with consortia of bacteria could help study interactions between them and thus identify mechanisms for malodor mitigation.

## Materials and methods

### Clinical study design

Two groups of subjects were recruited for this study —(1) 15 children consisting of 8 females and 7 males from 5 to 9 years old and (2) 15 teenagers consisting of 8 females and 7 males from 15 to 18 years old. At the start of the study (0 h), the subjects were given a standard bar soap (with no antimicrobial actives and no perfumes) to shower. One hour after shower, odor intensity (score range from 0 to 100) and character were evaluated at the underarm, back of the neck, and head scalp by an appointed professional perfumer. Microbiome samples were collected immediately afterwards for each site by tape stripping (D100 D-squame, CuDerm). Each tape strip was applied ten times against the skin with standardized pressure for 1 s and placed in 1.5 ml Eppendorf tube on ice. The subjects then participated in 4–5 h of vigorous exercise in an environment with a temperature of 32 °C and relative humidity of 66%. Briefly, the subjects were divided into groups to undertake a combination of physical activities (treadmill, indoor cycling, aerobics, and basketball) interchangeably under the directives of a professional instructor. The physical activities were carried out at intervals of 10–15 min with a break for 10–15 min. During the break, sweat samples were collected and the participants kept themselves hydrated. At the 8-h timepoint, odor intensity, odor character, and microbiome samples were collected again as described above (left or right underarm was randomly selected, but kept consistent across timepoints). Subjects were in an environment without air-conditioning throughout the 8 h period.

### Sample processing and DNA extraction

Tape strips were transferred into Lysing Matrix E tubes (MP Biomedicals), and 500 μL of ATL Buffer (Qiagen) was added. Samples were subjected to bead-beating with FastPrep-24 Instrument (MP Biomedicals) at a speed of 6.0 m/s for 40 s. Subsequently, samples were centrifuged at 16,000 *g* for 5 min and 200 μl of supernatant was treated with 10 μl of Proteinase K (Qiagen) and incubated at 56 °C for 15 min. DNA was extracted using EZ1 Advanced XL Instrument (Qiagen) with the EZ1 DNA Tissue Kit (Qiagen) with an elution volume of 100 μl and was quantified using Qubit dsDNA HS Assay Kit (Life Technologies) and later stored in a − 20 °C freezer.

### Library preparation, combinatorial pooling, and sequencing

A standard volume of 50 μl of the extracted DNA was subjected to shearing using Adaptive Focused Acoustics™ (Covaris). DNA libraries were prepared by using the Gene Read DNA Library I Core Kit (Qiagen) according to the manufacturer’s protocol except for the use of custom adapters in place of GeneRead Adapter I Set. Custom index primers were used for the enrichment of DNA libraries, which was performed according to an enrichment protocol adapted from Multiplexing Sample Preparation Oligonucleotide kit (Illumina). Libraries were quantified using Agilent Bioanalyzer, prepared with Agilent High Sensitivity DNA Kit or Agilent DNA1000 Kit (Agilent Technologies).

To avoid batch effects, whole-metagenome library pooling was performed in a combinatorial way (Additional file 2: Table S8). For the underarm, samples from children and teenagers were split evenly into two library preparation batches, with each batch containing an equal number of children, teenagers, higher malodor, and lower malodor samples. Libraries of samples from children were then pooled in equimolar amounts in one lane, and libraries of samples from teenagers in another lane. For the neck and head regions, libraries were built by preparing samples from the same subject (1 h and 8 h) one after another, alternating children and teenagers, as well as higher malodor and lower malodor samples. Samples from each collection site were multiplexed into one pool. Paired-end sequencing (2 × 101 bp reads) was done on successful DNA libraries using the Illumina HiSeq 2000 platform.

### Read pre-processing

After demultiplexing, reads were filtered using the Bcl2fastq software (CASAVA version 1.8.4) from Illumina, and FastQC was performed on the remaining reads (Pass Filter Reads). Sequencing bases with quality scores lower than 30 were trimmed off the 5′ and 3′ ends of each read, and reads with length less than 30 nucleotides were removed. Human reads were filtered out by mapping to the human genome (GRCh38) using BWA-MEM [40] (version 0.7.13-r1126; default parameters), leaving behind 4.71 billion non-human paired-end and singleton reads.

### Taxonomic classification

Taxonomic profiling of the samples was performed at all taxonomic ranks using MetaPhlAn2 [41] (version 2.5.0; default parameters). MetaPhlAn2 uses clade-specific markers genes covering bacterial, archaeal, viral, and eukaryotic microbes to perform unambiguous taxonomic assignments. The metagenome of each sample was analyzed independently to compute relative abundances. The output files were then merged into a single table listing the relative abundances per sample (columns) and per microbe (row). Taxa with mean relative abundance > 1% in either children or teenagers were conservatively identified for each body site for further statistical analysis.

### Functional profiling

HUMAnN2 [33] (v0.7.1; default parameters) was run with the UniRef90 protein database to derive functional profiles for each metagenome. HUMAnN2 provides (i) the abundance of each UnifRef orthologous gene family tabulated as reads per kilobase (RPK), (ii) abundance of MetaCyc pathways in RPK, and (iii) the coverage for each MetaCyc pathway, scaling from 0 to 1. Gene families and pathways with mean RPK values > 10 across samples either from children or teenagers were retained and were re-normalized to relative abundances for subsequent association analysis, while a MetaCyc pathway was only considered for further statistical analysis if the mean pathway coverage across all samples was > 0.3. Overall, we identified 69,792, 65,939, and 45,684 unique gene families from the metagenomes of the underarm, neck, and head samples, respectively. For MetaCyc pathways, there are 89 (underarm), 103 (neck), and 83 (head) pathways that met the above criteria. The percentage of reads assigned to gene families and pathways and those without assignment can be found in Additional file 2: Table S9. The contribution of each taxon to a gene family and pathway was also determined based on the taxonomic assignment of each read from HUMAnN2 outputs. Gene family contributions were visualized by mappings the UniRef90 IDs to KEGG Orthology group IDs in KEGG pathways [42] (version updated in February 2016).

### Statistical analysis

Multivariate analysis to evaluate the associations between odor intensity, age, and microbial communities before and after exercise was performed using the canonical analysis of principal coordinates approach (with Bray-Curtis dissimilarity) as implemented in the vegan R package (version 2.4-3). Significant associations between microbial taxa/functions and malodor were identified through a combination of statistical tests where (i) Spearman’s rho correlation coefficient between odor intensity and microbial taxa/functions was used for cross-sectional analysis and (ii) Wilcoxon signed-rank test of abundances before and after exercise was used for longitudinal analysis. *p*-values were adjusted using the Benjamini-Hochberg approach and all statistical analyses were performed in R. For pathways that were considered detected, association testing was done for youths, children, and teenagers to identify those that have FDR-adjusted *p*-values < 0.1 in at least two out of six comparisons as candidate pathways for further gene level analysis.

### Isolation of microbes from the axilla region of subjects

Microbes were aseptically collected from individuals in this study by swabbing the axilla region using pre-moistened (sterile 0.15 M NaCl + 0.1% Tween 20 solution) flock swabs (Puritan Med). A swab sample from the subject with the highest odor intensity was plated onto TSA blood agar (Thermo Fisher Scientific) and incubated at 37 °C overnight. Representative single colonies based on morphologies were selected and freshly sub-cultured for MALDI-TOF mass spectrometry analysis (Bruker) for species identification. Species identification was further verified by 16S rRNA sequencing (Charles River). Selected bacteria of interest (*Staphylococcus epidermidis*, *Staphylococcus hominis, Cutibacterium acnes*, and *Micrococcus luteus*) were then suspended in cryobeads (Thermo Fisher Scientific) and stored in a − 80 °C freezer.

### Sweat collection and culture experiments

Human sweat was collected from the underarm and neck area of multiple individuals in this study using 1.5 ml Eppendorf tubes, pooled in a 50 ml sterile falcon tube, and stored at 4 °C. The sweat secretion stock was then sterilized through 3000 *g* × 10 min centrifugation followed by 0.2 μm pore size sterile syringe filters (Millipore). Bacterial isolates were freshly prepared overnight and harvested the following day when OD_600_ reached 1.0. This was followed by centrifugation at 3000 *g* for 10 min, the resulting pellet was washed with sterile 0.01 M phosphate buffer (pH 6.0) and re-suspended in 10 ml bacteria-free sweat pool. These freshly prepared bacteria-sweat pool samples were incubated in orbital shaker aerobically at 120 rpm, 37 °C for 24 h. Every experiment included negative controls (10 ml filter-sterilized sweat pool without bacterial inoculation) under the same conditions as above. All experiments were conducted under aerobic conditions.

### Analytical detection for sour precursors and odorants

Leucine and isoleucine were measured by high-performance liquid chromatography (HPLC). Briefly, 0.2 ml of sample was pipetted into a 15 ml centrifuge tube. This was followed by the addition of 4.8 ml of 0.05 N hydrochloric acid and dispersion of the sample using vortex mixer. The resulting solution was filtered through a 0.45 μm PVDF syringe and mixed with 0.1 ml of sodium borate buffer (pH 10.2) and 0.2 ml of NBD-F solution in a 2 ml HPLC amber vial. After sealing the cap tightly, the vial was heated in an oven at 60 °C for 5 min, then cooled down before the addition of 0.4 ml of 0.05 N hydrochloric acid and injection to HPLC for analysis. The measurement of glycerol was performed using GC-FID. Five milliliters of sweat was lyophilized overnight, followed by the addition of 0.2 ml of water and 1.8 ml of extraction solvent into the tube. The sample was then filtered through 0.45 μm PVDF syringe into a GC vial for analysis.

To measure the levels of lactic acids in sweat, first, a calibration curve using 0.0, 0.25, 0.5, 1.0, 2.0, and 5.0 ppm of the lactic acid standard was established by monitoring the mass ion of m/z89 in the selected ion mode (SIM) in the LC/MS analysis (ABSciex 4000 QTRAP). Then, samples of sweat were diluted 500 times using methanol/water (1:1) and were injected into the LC/MS system for analysis and the peak areas of the mass number from lactic acid (m/z89) were obtained from the samples. The levels of lactic acid were determined by comparing peak areas from the samples and responses from the calibration curve.

The pH of the collected sweat sample (10 ml) was adjusted to 12.0 by the addition of NaOH (Wako), and lyophilization was performed for 2 days. After lyophilization, for each tube of residues, 1 ml of HCl (50% dilution with 37% HCI) (Sigma) was added, followed by vortexing and centrifugation at 4000 rpm and 20 °C for 20 min. The resulting supernatant was transferred into an Eppendorf tube, 150 μl of chloroform (Sigma) was added, followed by vortexing and centrifugation at 14800 rpm and 4 °C for 20 min. The chloroform fractions were then pooled together, and 10 μl of the pooled medium was injected into a GCMS instrument for analysis. An Agilent GC 7890B/MSD 5977A (Agilent, Wilmington, DE, USA) with Gerstel Autosampler MPS2 -TDU-CIS4 (Gerstel, Germany) was used for the separation and analysis of body odor compounds. The GC-MS was equipped with a DB-Wax column, 60 mm × 0.25 mm with 0.25 μm film thickness (Agilent, Delaware, USA). The large volume liquid injection mode was used—initial injector temperature was at 10 °C, equilibrated for 0.5 min, ramp of 12 °C/min was used to an end temperature of 220 °C, then held for 5 min. Injection volume was 10 μL. The oven temperature was held at 40 °C for 2 min, ramped to 220 °C at 3 °C/min and held for 4 min. The helium carrier gas flow was constant at 1.2 ml/min. The mass spectrometer was operated at an ionizing energy of 70 eV with a four scans/s rate over a range of *m*/*z* 29–450 and an ion source temperature of 230 °C. Identification of structures/compounds was performed using the National Institute of Standards and Technology Library (NIST’11) and an internal P&G MS library with commercially available standards. GC-olfactometry was performed at the initial run. Olfactory measurements were taken by the same professional perfumer who evaluated odor characteristics and intensity for the subjects in this study.

## Additional files

Additional file 1: **Figures S1-S10**, **Tables S1, S3 and S4**. (PDF 3276 kb)

Additional file 2: **Table S2.** Odor assessment and metagenomic sequencing data for all samples in this study. **Table S5.** GC-olfactometry of pooled sweat collected from children and teenagers. **Table S6.** Detected MetaCyc pathways that were found to be associated with malodor based on pathway abudance values from HUMAnN2. No significant association were detected for the head region. **Table S7.** Key pathways associated wth malodor production and their taxonomic contributors. **Table S8.** Information on how samples were distributed in library preparation and sequencing batches to avoid batch effects. **Table S9.** Reads mapped (%) to UniRef90 gene families and MetaCyc pathways. (ZIP 92 kb)

## Abbreviations

3M2H: 3-Methyl-2-hexenoic acid
3M3SH: 3-Methyl-3-sulfanylhexanol
HMHA: 3-Methyl-3-hydroxy-hexanoic acid
HPLC: High-performance liquid chromatography
LC/GCMS: Liquid chromatography and gas chromatography-mass spectrometry
