# Supplementary material for: Understanding the microbial basis of body odor in pre-pubescent children and teenagers
Source: Microbiome. 2018 Nov 29;6:213. doi: 10.1186/s40168-018-0588-z (PMC6267001; doi:10.1186/s40168-018-0588-z)
Supplement: Supplementary file 1 — Figures S1-S10, Tables S1, S3 and S4. (PDF 3276 kb) [file 40168_2018_588_MOESM1_ESM.pdf]

**Survey Subject**	Filipino mother with children & teenagers
**Climate**	Topical, hot & humid (average temperature = 27-32°C and relative humidity = 77%)
**Incidence of malodor**	High incidence (100%) across children and teenagers, as reported by their mothers
**Incidence of malodor per body part**	Underarm (98%), Neck (100%), Back (90%), Scalp (85%), Hair (82%), Feet (71%)
**Is malodor a source of concern?**	64% strongly agree and 36% agree across children and teenagers, as reported by their mothers
**Odor characteristics**	Mainly Sour
**Compensating hygiene habits**	Multiple baths per day.<br>(77% of subjects bathe 2-3 times per day and 91% bathe in the morning).

**Life Impact Questionnaire**	**% Agreement (Strongly agree or agree)**	**% Neutral (Neither agree nor disagree)**	**% Disagreement (Strongly disagree or disagree)**
I feel embarrassed or self-conscious when I have body odor	94	3	3
My body odor conditions have interfered with the quality of my work life, social life or family life	88	7	5
My body odor conditions have deterred me from engaging in social and leisure activities	80	8	13
My body odor conditions have prevented me from working or studying	68	12	20

Table S1: Summary statistics based on quantitative consumer habits & practices survey conducted by Procter & Gamble and Philippines FB consumer research services Inc, involving 300 families (150 families with 4-12 year old children, 150 families with 13-18 year old teenagers) in metro Manila area (July-August 2015).

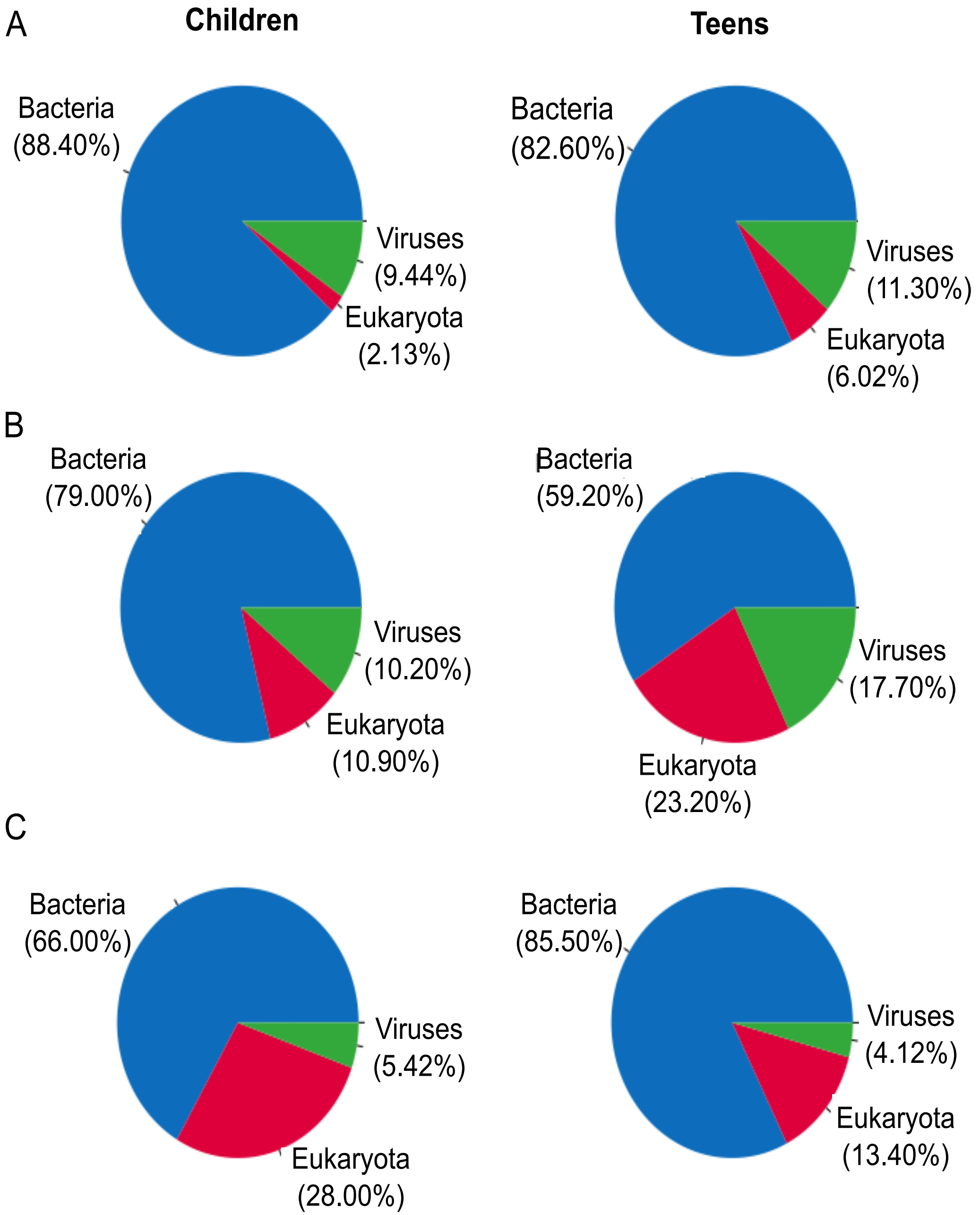

Figure S1: Pie-charts showing kingdom-level relative abundances in the skin microbiome at different body sites, (A) underarm, (B) neck and (C) head.

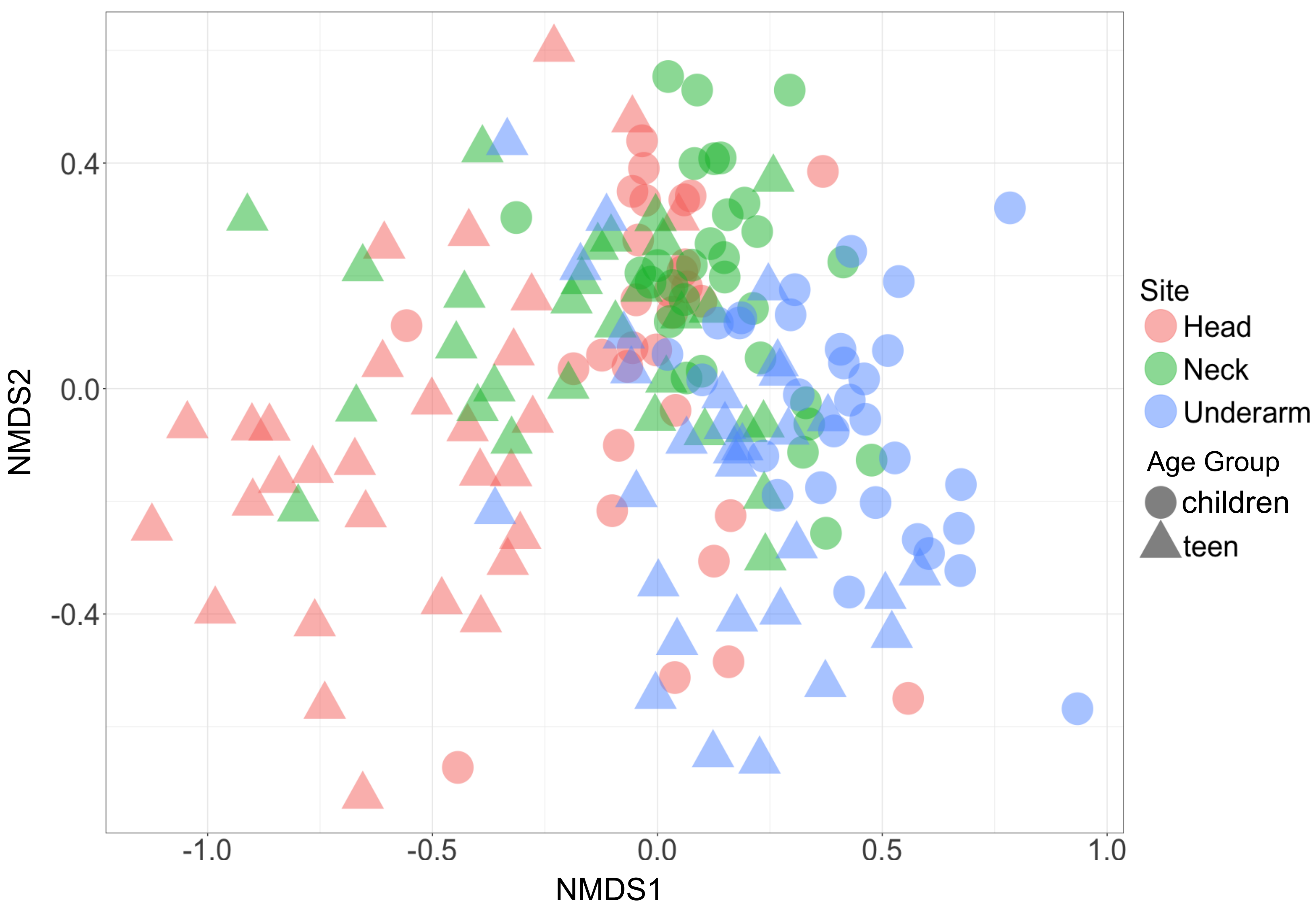

Figure S2: Non-metric multidimensional scaling plot of skin microbiomes across body sites using the Bray-Curtis dissimilarity.

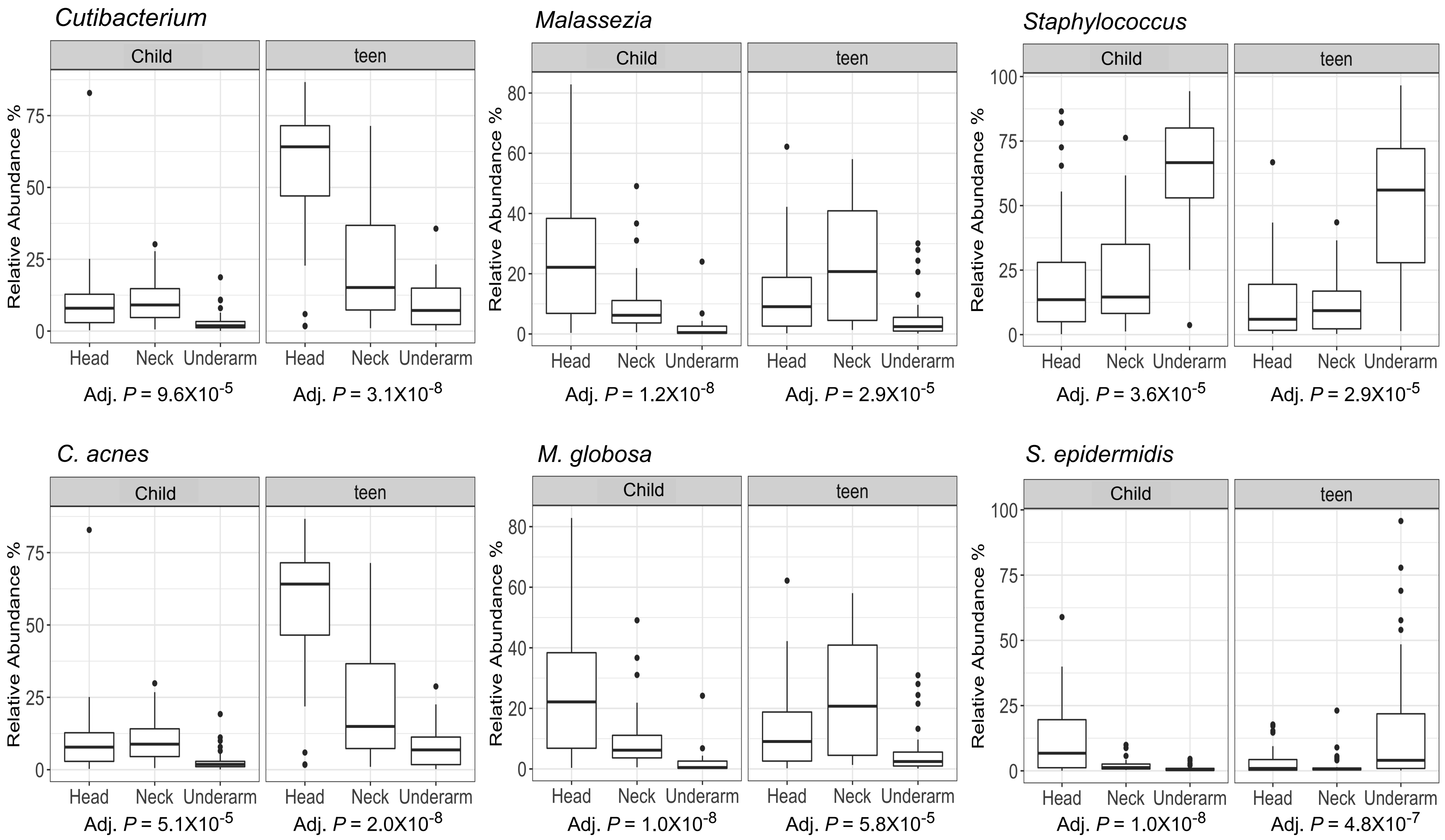

Figure S3: Identification of significant microbial genera and species associated with different body sites (head, neck and underarm) using a Friedman test (FDR-adjusted $P < 0.05$).

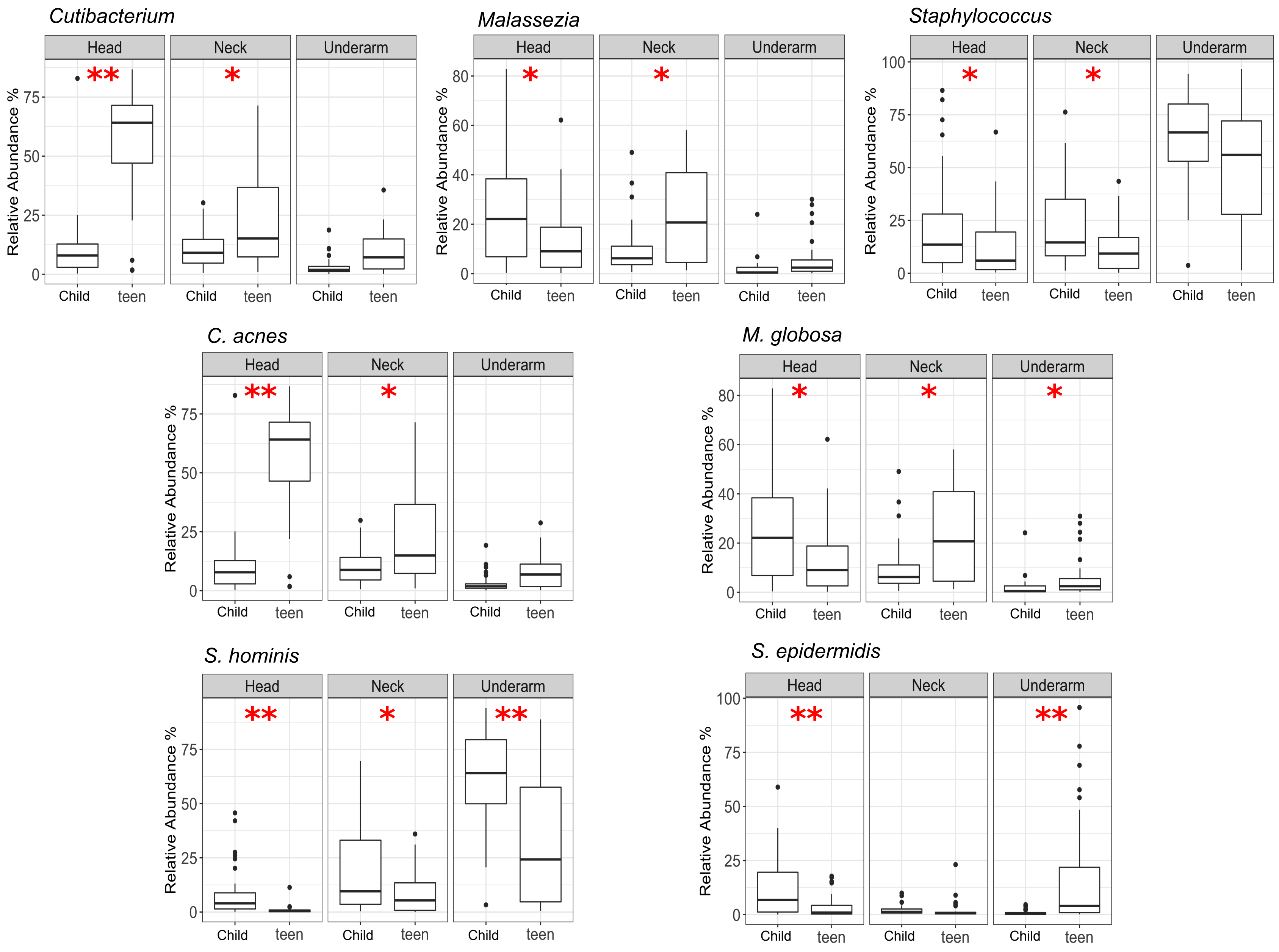

Figure S4: Identification of microbial genera and species associated with specific age groups (children and teenagers) using Wilcoxon signed rank test. ** = FDR-adjusted *P*<0.005; * = FDR adjusted *P* < 0.05

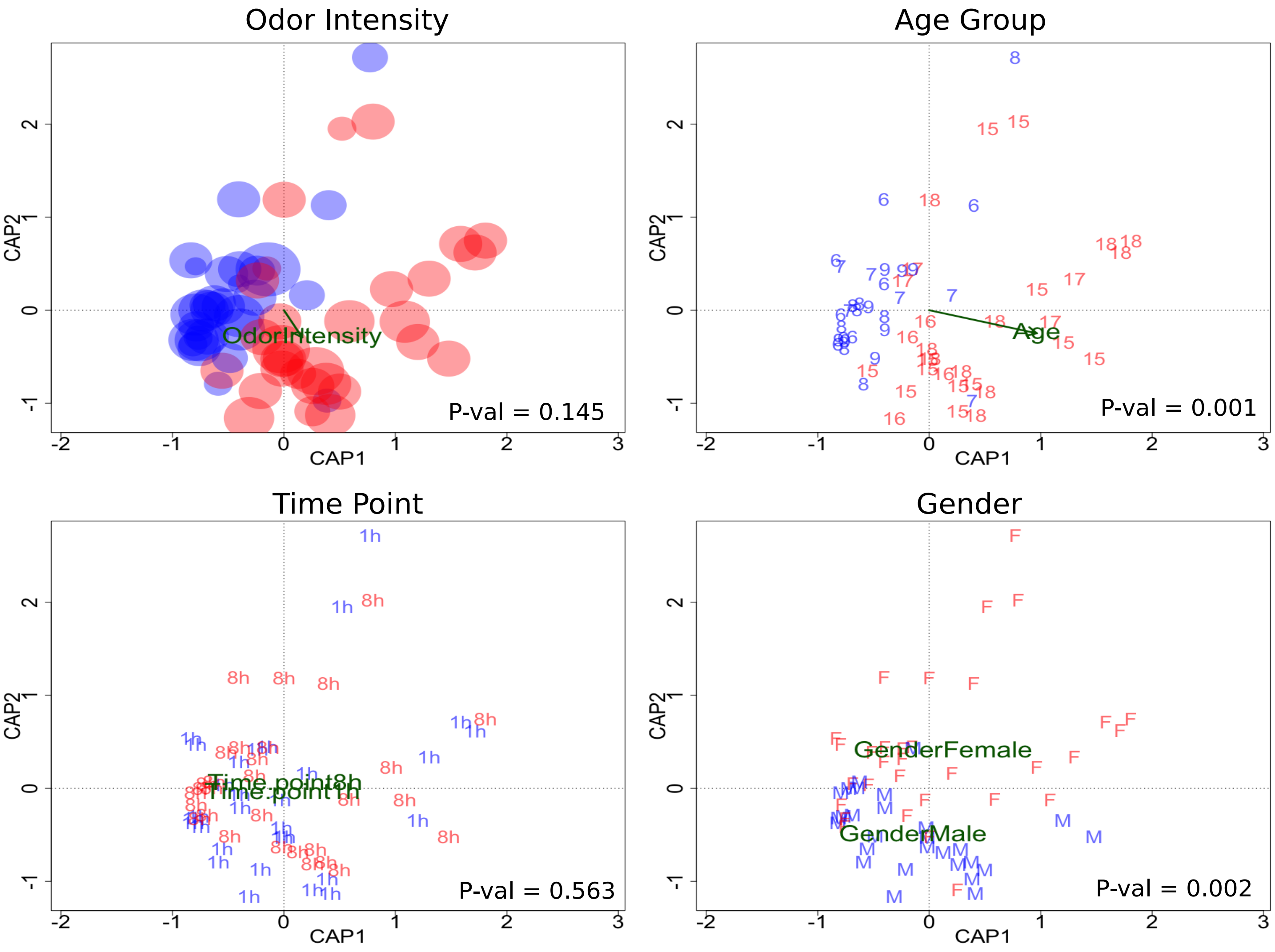

Figure S5: Ordination biplots for canonical analysis of principal coordinates (CAP) illustrating the strength of associations between odor intensity, age, sampling time and the skin microbiome on the scalp. CAP analysis is based on the Bray-Curtis dissimilarity. Size of each dot represents the relative odor intensity score in each sample (blue – children, red – teens, top left plot), while values indicating age (as a number), time point of sample collection (before [1h] or after [8h] exercise), and gender (**M**ale or **F**emale) are provided in corresponding subplots.

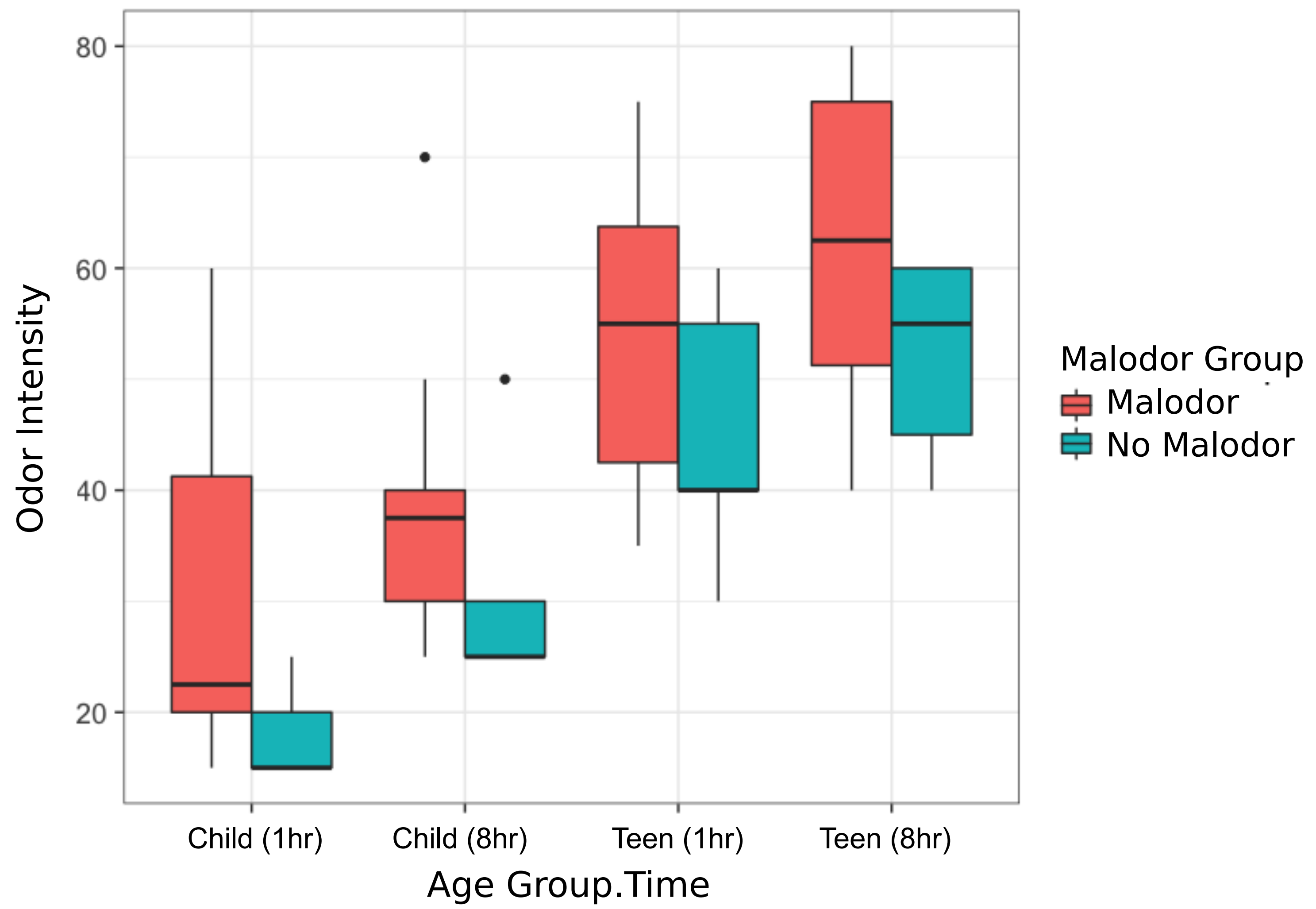

Figure S6: Differences in odor intensity across parent-defined malodor groups in children and teenagers at different timepoints. In all cases, "malodor" and "no malodor" groups were compared and found to not have a statistically significant difference in odor intensity (Wilcoxon test *p*-value > 0.1). Differences between children and teenagers were found to be significant in all cases (Wilcoxon test *p*-value < 0.05).

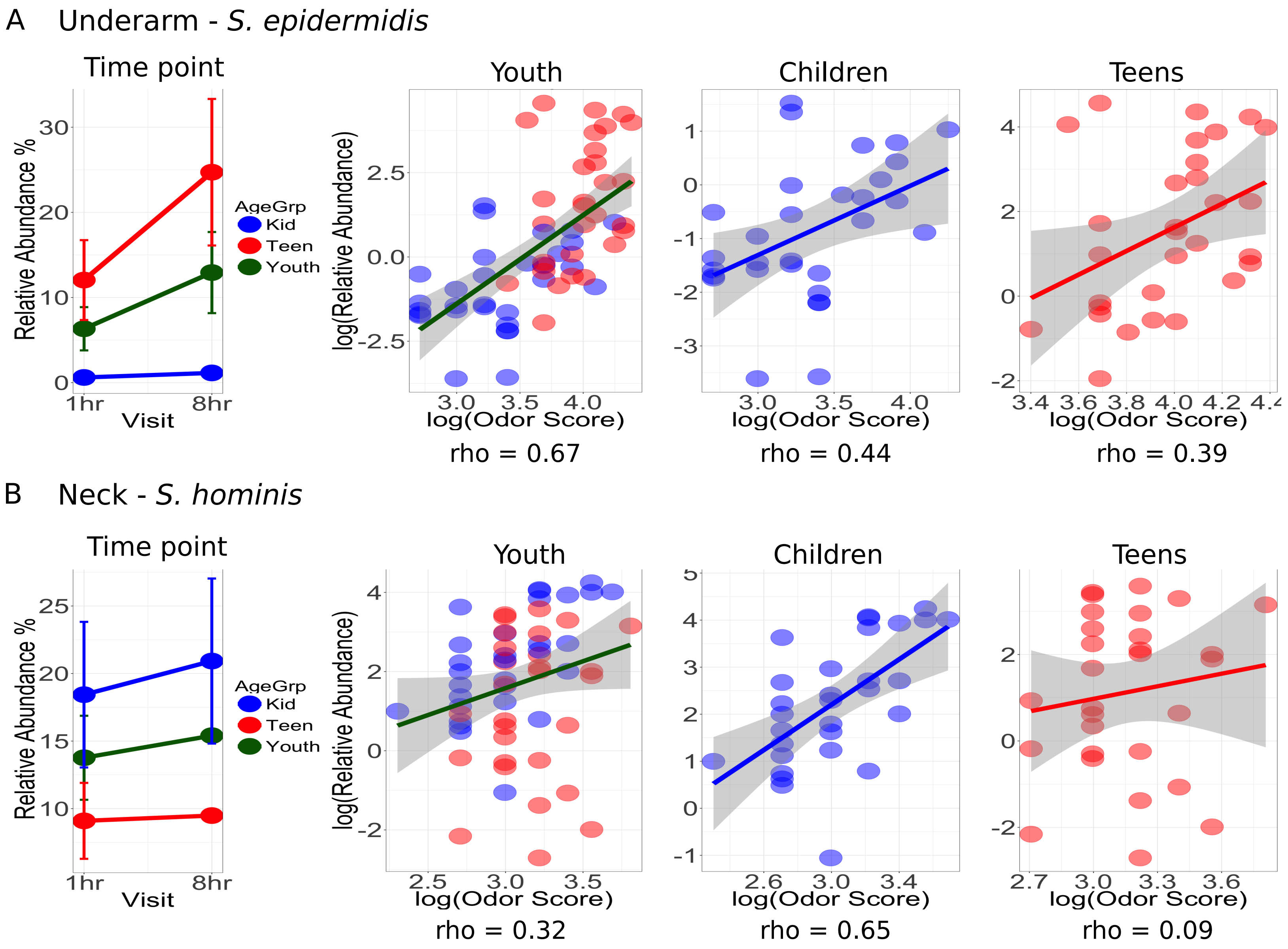

Figure S7: Correlation between odor intensity and *Staphylococcus* species in different body sites and across age groups. Interestingly, *S. epidermidis* shows strong correlation with odor intensity in the underarm in children and teenagers (and even stronger in the combined analysis – youth), but increases more notably with exercise in teenagers. *S. hominis* on the other hand is correlated with odor intensity primarily in the neck region of children and also increases in relative abundance with exercise.

		**Youth**				**Children**				**Teenagers**			
**Site**	**Genus**	Spearman ρ		Wilcoxon		Spearman ρ		Wilcoxon		Spearman ρ		Wilcoxon	
		ρ	p-value	p-value		ρ	p-value	p-value		ρ	p-value	p-value	
Underarm	*Malassezia*	0.53	10^{-3}	↓	n.s.	0.36	n.s.	↓	n.s.	0.33	n.s.	↑	n.s.
	Deinococcus	-0.39	0.087	↓	n.s.	-0.55	0.057	↓	n.s.	-0.10	n.s.	↓	n.s.
	Paracoccus	-0.42	0.057	↓	10^{-3}	-0.52	0.09	↓	0.027	-0.21	n.s.	↓	0.025
Neck	*Acinetobacter*	-0.43	0.057	↓	10^{-5}	-0.49	n.s.	↓	10^{-4}	-0.19	n.s.	↓	0.025
	Cutibacterium	-0.27	n.s.	↓	n.s.	-0.55	0.069	↓	n.s.	-0.20	n.s.	↓	n.s.
	Staphylococcus	0.35	n.s.	↑	n.s.	0.61	0.045	↑	n.s.	0.076	n.s.	↓	n.s.

Table S3: List of microbes at the genus level that show significant correlation with odor intensity in at least one age group. All reported *p*-values are adjusted for multiple hypothesis testing through FDR analysis (*p*-value < 0.1) and with Spearman ρ > 0.2. Arrows indicate directionality of change in relative abundance after exercise (up: ↑ or down: ↓).

Compounds	Children	Teenagers
Leucine	+	++
Isoleucine	+	++
Glycerol(Glycerin)	+	++
Lactic Acid	++	++

Table S4. Malodor precursors identified from sweat collected from children & teenagers, using high performance liquid chromatography (HPLC), gas chromatography with flame ionization detector (GC-FID) and LC-MS (see **Methods**). Legend: **+** = 0-5 PPM; **++** = 5-20 PPM; **+++** = 20 PPM and above (PPM=parts per million).

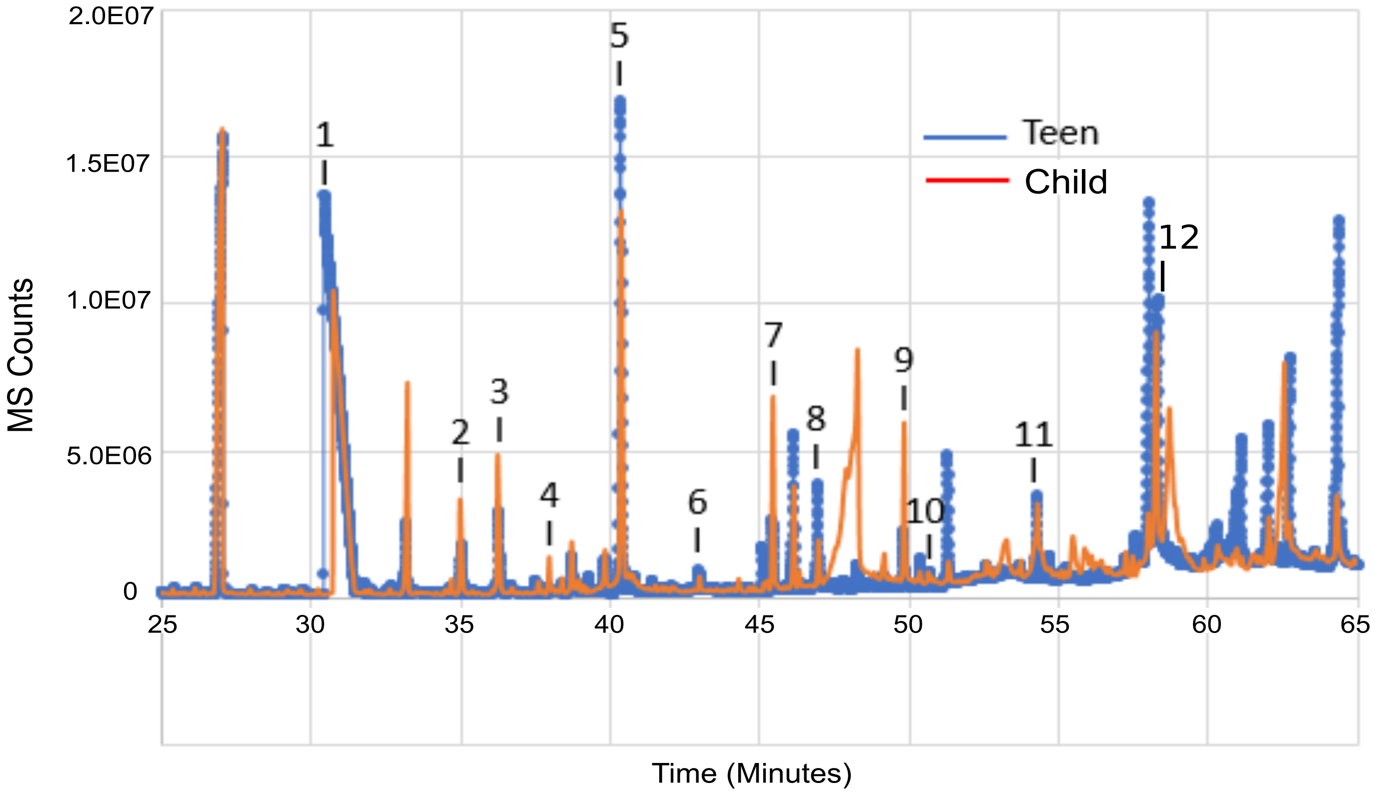

1. Acetic Acid
2. Propanoic Acid
3. 2-Methyl Propanoic Acid
5. Isovaleric acid &
   2-methyl-butanoic acid
6. Pentanoic acid
7. 4-Methyl Pentanoic Acid
8. Heptanoic Acid
9. 4-Methy-Hexanoic Acid
10. Heptanoic Acid
11. Octanoic Acid
12 Lactic Acid

Figure S8: GCMS profiles after 24hr incubation of pooled sweat collected from children & teenagers.

Valine, leucine & isoleucine biosynthesis

Children　　　　　　　　　　　　　　Teens

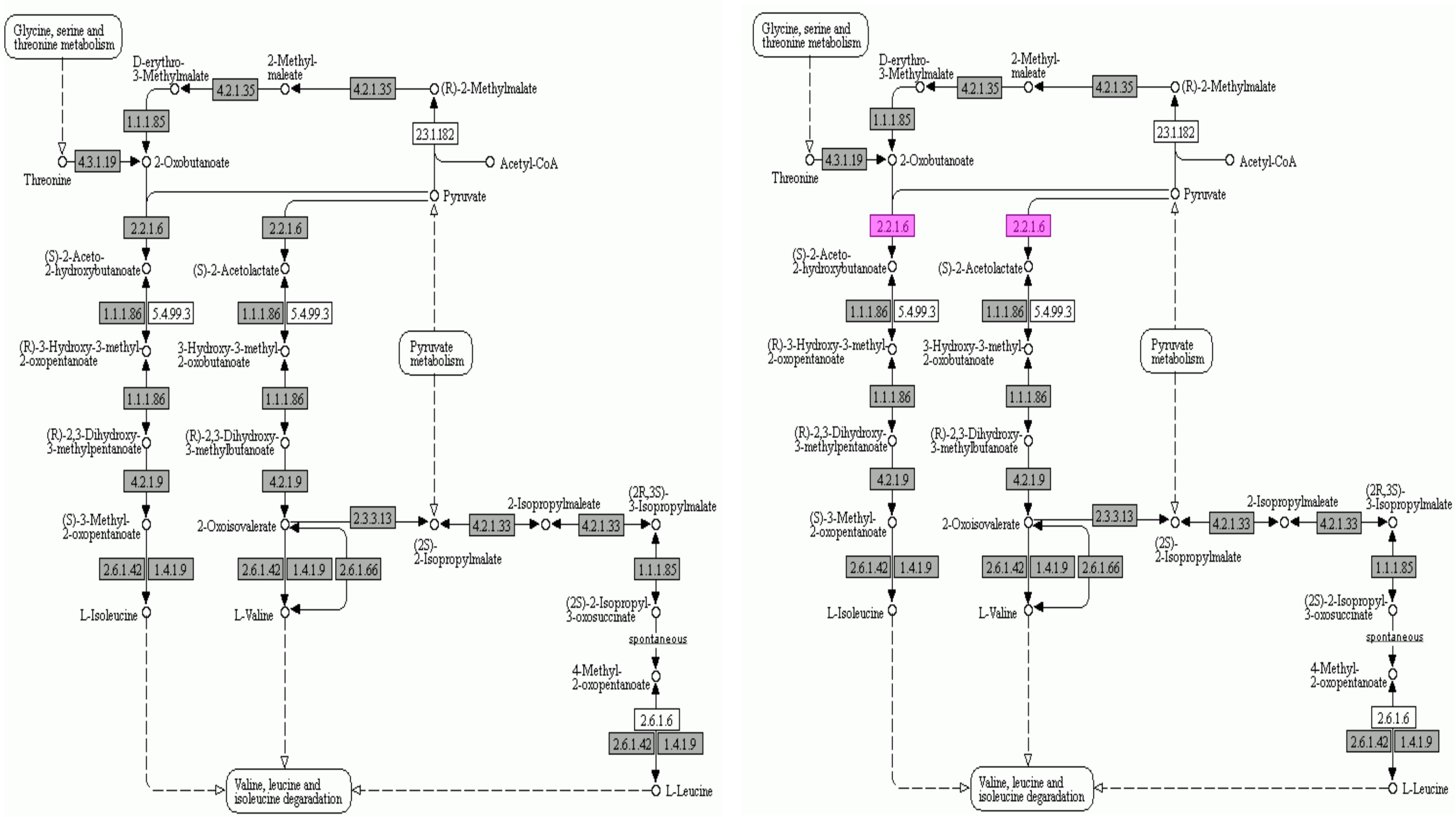

Figure S9: Correlations with odor intensity in the Leucine, Valine & Isoleucine biosynthesis pathway based on a “bag of genes” approach. Relative abundance of each enzyme (KO) was derived from the aggregated contribution of the whole metagenome and pink boxes indicate significant positively correlated KOs within the underarm metagenome in each age group, while grey boxes indicate no correlation with odor intensity. These results are in contrast with those seen in **Figure 3**, which is based on a “bag of genomes” approach and highlights the importance of this pathway for underarm malodor.

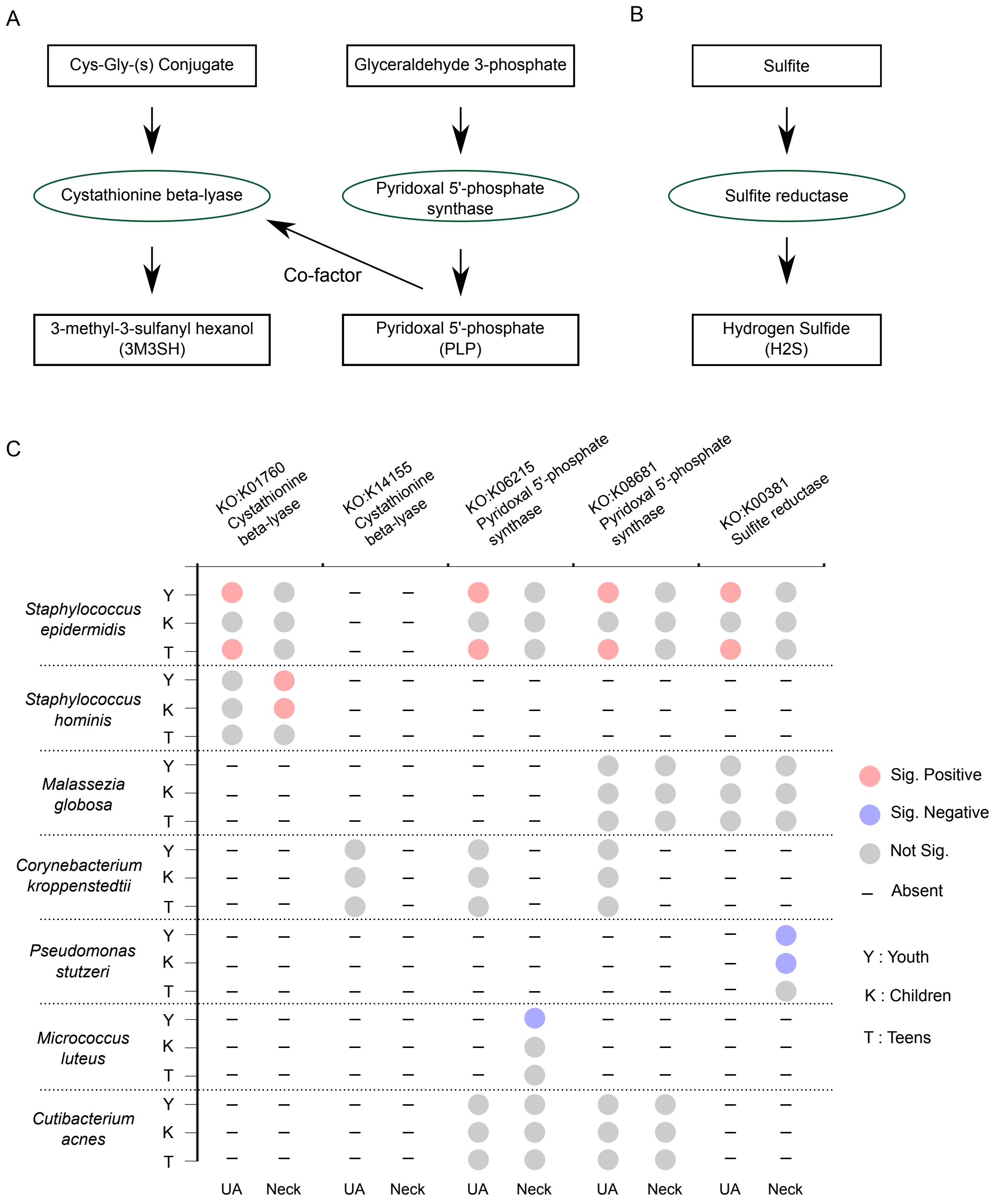

Figure S10: Representation of sulfur odor production pathways in the skin microbiome of children and teenagers. KEGG pathway diagrams for the production of (A) 3-methyl-3-sulfanyl hexanol, and (B) Hydrogen sulfide. (C) Representation of corresponding malodor-associated enzymes in different malodor-associated microbes. UA: Underarm, Sig: Significant.
